# Polygenic and Pleiotropic Adaptation of Yaks to High Altitudes: A Genome‐Wide Study of Hematological and Physiological Traits

**DOI:** 10.1002/age.70175

**Published:** 2026-08-11

**Authors:** Guangming Sun, Ziqi Liu, Hui Jiang, Xin Li, Hui Zhang, Ouzhuluobu, Xuebin Qi, Basangwangdui, Siyu Ge, Yiming Wang, Wangshan Zheng, Yanbin Zhu

**Affiliations:** ^1^ Institute of Animal Husbandry and Veterinary Medicine, Xizang Academy of Agricultural and Animal Husbandry Sciences Lhasa China; ^2^ Key Laboratory of Animal Genetics and Breeding on Tibetan Plateau, Ministry of Agriculture and Rural Affairs Lhasa China; ^3^ Kunming University of Science and Technology, Genetic Analysis and Sequencing Platform State Key Laboratory of Primate Biomedical Research (LPBR) Kunming China; ^4^ High Altitude Health Science and Translational Medicine Institute, Xizang Fukang Hospital Lhasa China; ^5^ School of Biological and Pharmaceutical Engineering Lanzhou Jiaotong University Lanzhou Gansu China

**Keywords:** *Bos grunniens*, genome‐wide association study (GWAS), physiological trait, Qinghai–Xizang plateau, whole genome sequencing, yak

## Abstract

Hematological adaptation is critical for oxygen transport at high altitudes. While hemoglobin concentration is well‐studied, yak ( *Bos grunniens* ) hematology versus lowland cattle remains under‐explored. We analyzed blood phenotypes from 1244 plateau yaks and 154 lowland cattle. Yaks exhibited elevated hemoglobin, hematocrit, and lymphocyte counts, but reduced platelet counts and monocyte/neutrophil percentages among other parameters, indicating distinct hypoxia adaptation. Using genome‐wide association studies (GWAS) and composite of multiple signals (CMS), we identified 6485 selection‐associated single‐nucleotide variants (SNVs) linked to 43 hematological traits (average 6.85% variance explained). These included 172 yak‐selected genes (YSGs), with *SLIT3*, *FSTL5*, and *PCDH15* showing top pleiotropic effects (*SLIT3* regulates immune function and oxygen transport). Thirty‐eight YSGs influenced multiple traits, and six (*SLIT3*, *FSTL5*, *PCDH15*, *PRIM2*, *CDH13*, *HLA‐DQB1*) overlap with genes under selection in Tibetan highlanders. Crucially, unlike hemoglobin attenuation in humans and other species, yaks show elevated hemoglobin, suggesting an alternative adaptive strategy. Cross‐species analysis revealed 76 YSG orthologues affect 13 human blood traits, indicating highland species are likely to have selected the same set of genes for hypoxia adaptation. Our findings demonstrate polygenic, pleiotropic mechanisms underpinning yak adaptation, providing a genomic framework for high‐altitude biology and conservation.

## 1 Introduction

Tibetan populations (Qi et al. 2013; Storz 2021; Zheng et al. 2023), along with high‐altitude livestock (Li et al. 2013; Ma et al. 2019; Liu et al. 2023) and indigenous animals (Liu et al. 2022), form “advantaged populations” for studying high‐altitude adaptation. Yaks ( *Bos grunniens* ), as a unique large mammal native to the plateau, possess exceptional adaptive traits that distinguish them as one of the most valuable bovine genetic resources. Current consensus suggests that extant wild and domesticated yaks share a common ancestor, with yaks having migrated to the Qinghai–Tibet Plateau around 5 million years ago, gradually adapting to its hypoxic and cold climate (Qiu et al. 2012; Gao et al. 2022; Liu et al. 2023). Domesticated yaks were tamed approximately 6000 years ago and are now primarily distributed across the Qinghai–Tibet Plateau and adjacent high‐altitude regions at elevations of 3000–5000 m (Qiu et al. 2015; Liu et al. 2023), playing an irreplaceable role in the livelihoods of local herders and the maintenance of the plateau's ecosystem.

Domestic yak have adapted to the plateau environment through multi‐system physiological changes, including increased respiratory rate, enhanced lung function (Gao et al. 2022), higher red blood cell count (Ma et al. 2019), dense fur (Qiu et al. 2012; Liu et al. 2023), and a lower metabolic rate (Qiu et al. 2015). In past decades, numerous studies have focused on uncovering the genetic basis of hypoxia adaptation in yaks, identifying a range of genes associated with high‐altitude adaptation (Qiu et al. 2015; Zhang et al. 2021; Gao et al. 2022; Liu et al. 2023). It has been found that yaks exhibit expansions in gene families related to sensory perception and energy metabolism compared to cattle, with genes under natural selection primarily linked to hypoxia and nutritional metabolism (Qiu et al. 2012). Moreover, introgression and structural variations have been identified as significant genetic factors contributing to the adaptation of yaks to high‐altitude environments (Liu et al. 2023).

The hematological system is one of the most critical systems for oxygen transport and nutrient exchange (Gilbert‐Kawai et al. 2014; Yang et al. 2016; Storz 2021), with hemoglobin concentration being a hallmark phenotype of high‐altitude adaptation. The *EPAS1* gene, which regulates hemoglobin levels, is considered one of the key regulatory genes for high‐altitude adaptation (Peng et al. 2017). However, research on yak hematological phenotypes remains fragmented, and the patterns and genetic mechanisms underlying these phenotypic changes are yet to be fully elucidated. In this study, we systematically analyzed the blood phenotype variation patterns and genetic basis in high‐altitude yaks and lowland migratory cattle, providing essential genetic evidence for the study of adaptive phenotypes and the elucidation of genetic mechanisms in yaks.

### 1.1 Identification of Yak Adaptive Blood Traits

The domestic yaks were well adapted to the high‐altitude environment, reflected across multiple organ systems. Here, we collected blood phenotypic data from 1398 high‐altitude cattle (including 1244 yaks and 154 lowland‐migrated cattle), covering 43 phenotypic traits (23 hematological and 20 biochemical parameters). After rigorous quality control (QC), 1029 individuals (895 yaks and 154 lowland‐migrated cattle) were included in the subsequent analyses. To identify the patterns of variation in 43 blood phenotypes within yaks (Table S1). We conducted a phenotypic correlation analysis. The results indicate that these phenotypes can be categorized into modules such as oxygen transport (e.g., hemoglobin), coagulation function, electrolyte balance, liver function, and kidney function, consistent with physiological and biochemical blood assessments (Figure S1).

**FIGURE 1:**
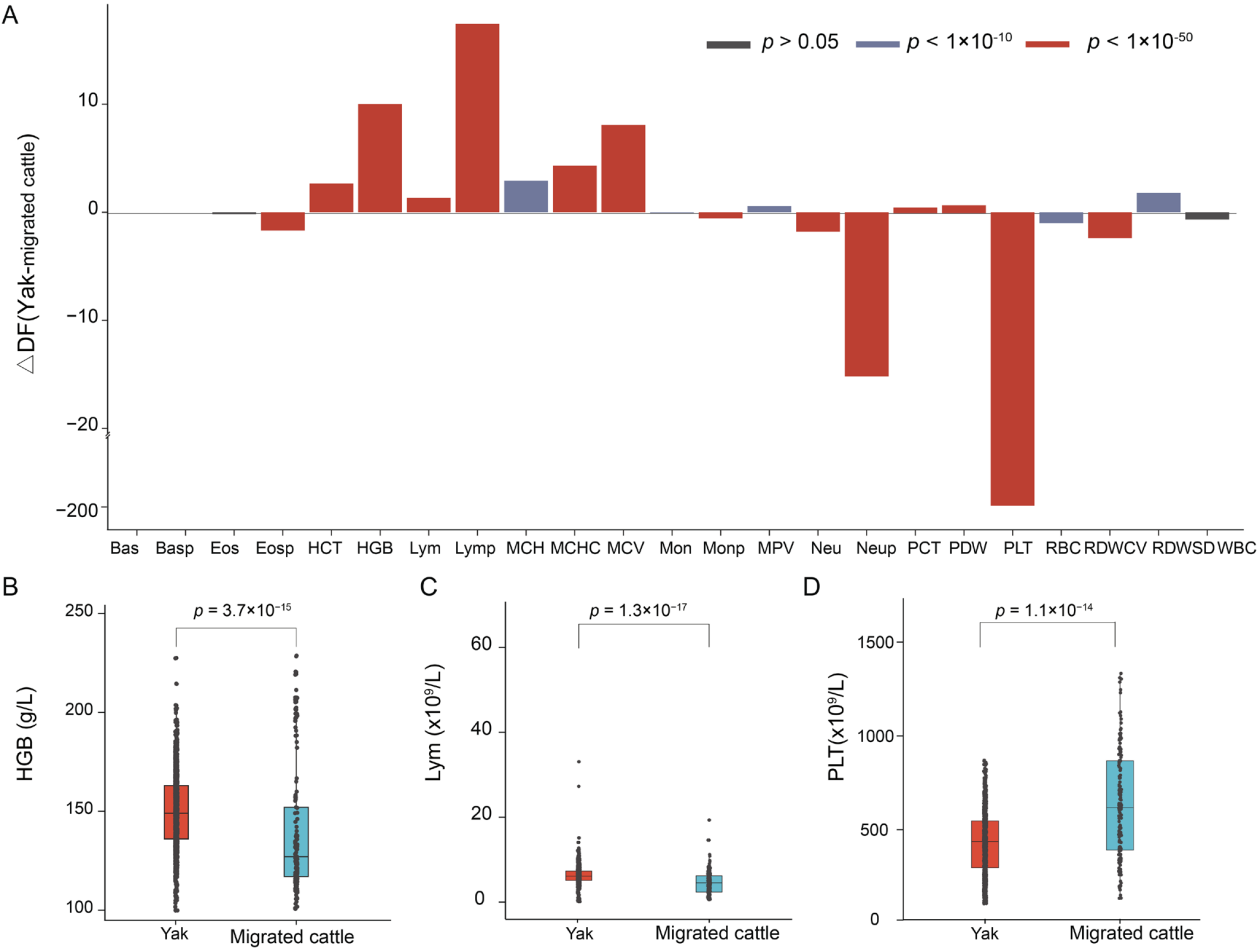
The differential analysis of 23 traits in yaks. (A) Direction of blood phenotype differences, where we represent the mean difference (△DF, yak—migrated cattle) to indicate the direction of the phenotype difference. The magnitude and sign of the difference reflect the direction, with the significance of the differences tested using a *T*‐test and *p*‐values adjusted using the false discovery rate (FDR). (B) Hemoglobin concentration (HGB); (C) Lymphocyte count (Lym); (D) Platelet count (PLT).

The direction of phenotypic differences likely reflects the trajectory of high‐altitude adaptation (He, Zheng, et al. 2023). We performed differential analysis using lowland‐migrated cattle as controls, with age and sex as covariates. Among the 23 hematological traits, 11 phenotypes were significantly higher in yaks compared to lowland‐migrated cattle (e.g., HGB, HCT, PDW), 8 were significantly lower (e.g., Mon%, PLT, RDWCV), and 4 showed no significant difference (Figure 1A, Table S2). We observed that the directional changes in several phenotypes were consistent with those seen in Tibetan populations (e.g., RDWCV) (He, Zheng, et al. 2023) (Figure 1C). Additionally, we identified several classical phenotypes whose directional changes were inconsistent with those observed in Tibetan populations and other high‐altitude species (e.g., HGB, Lym) (Figure 1B,D, Figure S2, Table S2). The attenuation of hemoglobin concentration is widely recognized as a hallmark of high‐altitude adaptation across various species. However, our phenotypic differential analysis revealed that hemoglobin levels in yaks were significantly higher than in lowland‐migrated cattle (Figure 1B, Figure S2, Table S2), with similar trends observed in lymphocyte counts. These distinct variations be likely to represent specific phenotypic adaptation patterns in yaks to the high‐altitude environment.

### 1.2 Genome Whole Association Study of 43 Yak Blood Trails

**FIGURE 2:**
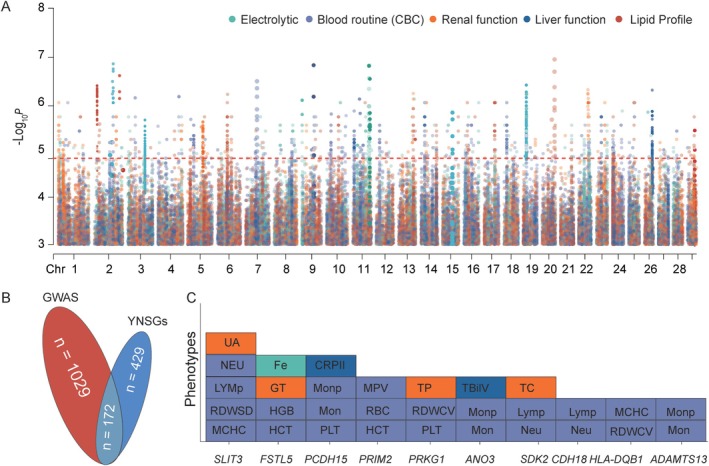
Genetic associations of the scanned traits in Yaks. (A) The Manhattan plot illustrating the GWAS signals of 43 traits in Yak. Only the top1 signal of each trait was integrated into this Manhattan plot for visualization, and the detailed information is available in Table S4. The significant threshold (*p* < 1 × 10^−5^) is denoted by the red line. (B) 172 genes associated with yak blood phenotypes that are under natural selection (the Venn diagram illustrates the overlap between GWAS signals for yaks and genes related to high‐altitude adaptation under natural selection); (C) the top 10 genes associated with multiple blood phenotypes.

To identify the genetic basis of adaptive phenotypic variation in yaks, we performed whole‐genome sequencing on 129 unrelated samples (114 yaks and 15 lowland‐migrated cattle) and detected genomic variants using the well‐trained GATK 4.0 standard pipeline. We identified 32 514 937 variants (including 27 180 326 single nucleotide variants and 5 334 611 insertions/deletions) (Methods in Section 3 and Figure S3A). After stringent QC, 107 individuals and 12 855 162 variants were included in the genome‐wide association studies (GWAS) (Methods in Section 3 and Figures S3B and S4). Using gender and age as covariates, we conducted GWAS for 43 quantitative traits (23 hematological and 20 blood biochemistry traits) using the GEMMA linear mixed model, with the significance of genetic associations evaluated by the likelihood ratio test (LRT) *p*‐value. Given the sample size limitations, a genome‐wide significance threshold of *p* < 1 × 10^−5^ was applied. We identified 1697 genomic regions associated with various blood phenotypes in yaks, with 668 of these regions located in intergenic areas (Figure 2A, Table S3 and Figures S5–S9).

For GWAS, in total, we identified 6485 SNVs with genome‐wide significant associations (*p* < 1 × 10^−5^) among 43 traits, and these variants are located in 879 independent genomic regions (see Methods in Section 3). Of these, there are 7 signal regions regarding 3 traits previously reported (Table S4) (Ma et al. 2019), and the other 872 signal regions regarding 40 traits are newly identified GWAS signals (the signals represent at least one SNV within a 1 Mb window) (Table S3). The top 3 signals include a variant (13:67022078) in the *POU2AF2* gene associated with gamma‐glutamyl transferase (GT, *p* = 1.03 × 10^−7^), a variant (16:40225508) in the *FSTL5* gene associated with gamma‐glutamyl transferase (GT, *p* = 3 × 10^−7^) and hemoglobin (HGB, *p* = 1.0 × 10^−6^), and a SNV (4:7979720) in the *GIMAP1* gene associated with Ca (*p* = 7 × 10^−7^) (Table S3).

To see how many of the associated variants/genes are also the targets of putative nature positive selection in Yaks, the composite of multiple signals (CMS) was employed to identify a high‐confidence list of variants/genes with confirmed selective signals based on the genome sequencing data, including 67 000 Yak selected SNVs (YSSs) and 429 Yak selection genes (YSNGs). The results indicate that 6637 candidate selective single nucleotide variants (YSSs) are associated with 43 traits, encompassing 172 genes (Table S5). Among 172 genes, 38 genes are associated with multiple traits, meaning a single gene is associated with multiple traits (Figure 2A and Table S5). The top 10 genes include *SLIT3*, *FSTL5*, *PCDH15*, *PRIM2*, *PRKG1*, *ANO3*, *SDK2*, *CDH13* and *HLA‐DQB1*. *SLIT3* displays the highest pleiotropy, being associated with five traits: MCHC, RDWSD, LYM, NEU, and UA. This suggests that *SLIT3* may be closely related to yak renal function (UA), immune function (NEU and LYM), and oxygen transport (MCHC and RDWSD) (Figure 2C). Notably, six genes (*SLIT3*, *FSTL5*, *PCDH15*, *PRIM2*, *CDH13*, and *HLA‐DQB1*) have been reported to be under strong positive selection in Tibetan populations, contributing to high‐altitude adaptation (Zheng et al. 2023). This suggests that yak may have enriched a cluster of genes to regulate hemoglobin, renal function, and liver function, aiding their adaptation to high‐altitude environments.

To quantify the genetic contribution of YSGs to phenotypic adaptation in yaks, we next estimated the proportion of variance in phenotypic differences explained (PDE) by each YSG (Methods in Section 3). In general, 172 YSGs explain 43 traits with an average PDE of 6.85%. The highest PDE is 16.58% for *RGS1*, accounting for the adaptive trait of lymphocyte percentage (Lym%), three genes involved in the regulation of hemoglobin, explaining 14.58% of the phenotypic variance in hemoglobin levels. Notably, when mapping the 172 natural selection‐related genes (YSGs) to the human genome, we found that 76 signal regions were significantly associated with 13 human blood traits (GWAS Catalog, 2025‐10‐10), suggesting that yaks may harbor a set of genes to regulate hemoglobin and maintain blood homeostasis. Together, our GWAS analyses established an atlas of the phenotype–genotype associations in yaks. We observed both polygenic and pleiotropic effects of genes contributing to the genetic adaptation of yaks.

## 2 Discussion

Yaks have exhibited remarkable adaptation to high‐altitude environments, with long‐term natural selection shaping their unique adaptive phenotypes. In this study, we systematically analyzed yak blood physiological and biochemical phenotypes along with genomic data, identifying 19 differential phenotypes and 172 selection‐related genes (YSGs). This analysis provides valuable insights into the blood phenotype changes and genetic mechanisms underlying yak adaptation to high‐altitude environments.

Among the 43 yak phenotypes analyzed, 20 were biochemical; due to incomplete control group biochemical data, we conducted differential analysis on only 23 blood physiological phenotypes. Significant differences were found in 19 phenotypes between yaks and lowland migratory cattle, with the most notable differences observed in lymphocyte percentage (Lym%) and platelet count (PLT). This suggests that yaks may enhance blood circulation and immune function to mitigate the effects of hypoxia. Notably, yaks exhibit higher hemoglobin concentrations than other high‐altitude mammals, such as humans (Wu and Kayser 2006; Peng et al. 2017; Zhang et al. 2017), Tibetan mastiffs (Gou et al. 2014), and Tibetan horses (Liu et al. 2019), which is consistent with previous studies. Thus, we hypothesize that elevated hemoglobin concentration is an alternative strategy by which yaks adapt to high‐altitude hypoxia.

By integrating genome‐wide association studies (GWAS) and natural selection signal scans, we identified 6637 selected SNVs significantly associated with 43 traits, involving 172 selected genes. Among these, 29 genes were involved in monocyte (Mon) regulation, explaining 2.98% of the genetic variance (Table S6). Eight genes were significantly associated with lymphocyte percentage, with each gene explaining an average of 7.53% of the phenotypic variance, accounting for 60.32% of the total phenotypic variance. Additionally, we identified two genes significantly associated with hemoglobin concentration, each explaining 12.55% of the phenotypic variance, indicating that blood phenotype regulation involves multiple genes and is in accordance with studies on high‐altitude adaptation in Tibetan populations (He, Guo, et al. 2023). We recognize that hematological traits exhibit inherent physiological interdependencies. Consequently, observed instances of genetic pleiotropy where single genes influence multiple phenotypic outcomes may reflect these intrinsic correlations rather than independent biological effects. Rigorous functional validation through targeted experiments will be essential to distinguish causal mechanisms from correlative associations, constituting the focus of our ongoing investigation.

*EPAS1* (Wu and Kayser 2006; Peng et al. 2017; Storz 2021) and *EGLN1* (Xiang et al. 2013) are well‐known genes associated with high‐altitude hypoxia adaptation, particularly *EPAS1*, which has been widely reported to regulate hemoglobin levels (Peng et al. 2017; Zhang et al. 2017). However, in this study, we did not detect any positive selection signals for *EPAS1* or its association with hemoglobin. This is likely due to the limited sample size in our study.

We acknowledge limitations in the current phenotypic dataset, including the absence of detailed cardiopulmonary function metrics and comprehensive anthropometric measurements. Additionally, the genomic analysis was constrained by a modest sample size (*n* = 102). Future studies should incorporate multi‐omics data, including expanded genomic, epigenomic, and high‐resolution phenotypic profiles, across major yak‐producing regions at varying altitudes. This integrated approach will enable multidimensional dissection of the physiological adaptations to high‐altitude hypoxia and their underlying genetic architecture in yaks.

Finally, The GWAS landscape of Yaks blood phenotypes considerably expanded the atlas of phenotype–genotype associations in domesticated animal living in an extreme environment, which will improve our understanding of phenotypic plasticity and genetic adaptability under strong natural selection.

## 3 Materials and Methods

### 3.1 Sample Collection

We collected phenotypic traits and blood samples from subjects (1244 yaks and 154 lowland‐migrated cattle) who lived in Linzhou counties of the Qinghai–Tibetan Plateau, China.

All experimental procedures involving yak and lowland cattle were conducted in strict accordance with the principles outlined in the Declaration of Helsinki (2013 revision). Animal studies adhered to the ARRIVE Guidelines 2.0. This research received formal ethical approval from the Institutional Review Board of Kunming University of Science and Technology (Approval: PZWH‐K2023‐0069) and the Animal Ethics Committee of Kunming University of Science and Technology. Continuous ethical oversight was maintained throughout the study duration.

### 3.2 Genomic Variant Calling and Filtration

Whole‐genome sequencing was conducted for 129 samples (114 yaks and 15 lowland‐migrated cattle) on Illumina platforms (BGI, generating 150‐bp paired‐end reads). Raw sequencing reads were subjected to quality assessment using FastQC (v0.11.9), followed by adapter trimming with Trimmomatic (v0.39). The resulting high‐quality reads were subsequently aligned to the domestic yak reference genome (assembly LU_Bosgru_v3.0, accession GCA_005887515.1) using BWA‐MEM (v0.7.17). Following alignment, PCR duplicates were identified and flagged using GATK MarkDuplicates (v4.2.6.10), although they were retained in the analysis. Base quality scores were then recalibrated using GATK BaseRecalibrator (v4.2.6.1).

Variant calling was performed individually per sample using GATK HaplotypeCaller (v4.2.6.1) in GVCF mode (emit reference confidence). These per‐sample GVCFs were integrated for joint genotyping using GATK GenomicsDBImport and GenotypeGVCFs (v4.2.6.1). This initial joint calling produced 32 514 937 raw variants, comprising 27 180 326 single nucleotide variants (SNVs) and 5 334 611 insertions/deletions (indels). Due to the lack of a yak‐specific high‐confidence variant set required for Variant Quality Score Recalibration (VQSR), a hard‐filtering approach was implemented using empirically optimized thresholds. SNVs were filtered based on the following criteria: QD < 2.0, FS > 60.0, SOR > 3.0, MQ < 40.0, MQRankSum < −12.5, or ReadPosRankSum < −8.0. Indels were filtered using thresholds of QD < 2.0, FS > 200.0, or ReadPosRankSum < −20.0.

### 3.3 Quality Controls

#### 3.3.1 Phenotype Quality Controls

Phenotypic Data Quality Control: For the 43 phenotypes, we implemented the following quality control (QC) measures: (1) Manually excluded individuals with erroneous records; (2) Removed individuals whose data deviated more than mean ±3 SD (if a sample's traits value is greater than the average value plus three times the standard deviation, or less than the average value minus three times the standard deviation, such an individual will be removed.); and (3) Excluded individuals younger than 1.5 years or older than 6 years.

#### 3.3.2 Genomic Data Quality Control

Genomic data QC was conducted following the published protocol (Anderson et al. 2010), and PLINK2 (Alpha 7.1) was employed to conduct both per‐individual and per‐SNV quality control.

The per‐individual QC included four steps: (1) exclusion of individuals with discordant sex information between genotype and recorded sex; (2) exclusion of individuals with missing genotype data and outlier heterozygosity rates, specifically a genotype failure rate (GFR) ≥ 0.05 and heterozygosity rate deviations of more than ±3 standard deviations from the mean; two individuals were removed; (3) exclusion of duplicated or related individuals, defined as a pairwise identity by descent (IBD) value > 0.1875; and (4) exclusion of individuals with divergent ancestry; five individuals were removed.

The per‐SNV QC included three steps: (1) exclusion of SNVs with excessive missing genotype rates (MGR > 0.05); (2) exclusion of SNVs showing significant deviation from Hardy–Weinberg equilibrium (HWE < 1 × 10^−6^); and (3) exclusion of SNVs with low minor allele frequency (MAF < 0.05).

Finally, 119 individuals (104 yaks and 15 land‐migrated cattle) and 12 855 162 variants were included for the subsequent analysis.

### 3.4 GWAS Analysis

We conducted genome‐wide association studies (GWAS) for 43 quantitative traits using a linear mixed model approach implemented in GEMMA (Zhou and Stephens 2012), which controls for both population structure and genetic relatedness through a relatedness matrix rather than principal components (PCs) of population structure. The significance of genetic associations was assessed using *p*‐values from the likelihood ratio test (LRT) generated by GEMMA.

### 3.5 Genome‐Wide Natural Selection Signal Scanning

To identify Yak selected single nucleotide variants (YSSs), we calculated composite of multiple signals (CMS) scores (Grossman et al. 2010) for genome‐wide variants using whole‐genome sequencing (WGS) data from 104 yaks and 15 migrated cattle.

For the Composite of Multiple Signals (CMS) calculation, we employed four distinct metrics as input parameters: two frequency‐based measures (*F*_ST_ and △*π*)and two haplotype‐based methods (iHS and XPEHH). The CMS score was calculated using the formula:

$$\mathrm{CMS}=-\log\prod_{i}p_{i}$$

where *i* denotes the *i*‐th single nucleotide variant (SNV) and *p*_*i*_ represents the transformed *p*‐value for that SNV derived from each of the four metrics, following the approach established by Zheng et al. (2023).

Prior to XPEHH and iHS computation, genome‐wide phasing was performed on data from 104 yaks using Shapeit4. Genetic distances for the XPEHH and iHS analyses were based on a conversion rate of 1 centiMorgan (cM) ≈ 1.03 megabases (Mb). Genome‐wide scans for XPEHH and iHS scores were conducted using Selscan (Szpiech 2024), with scores subsequently standardized across the genome. SNVs exhibiting an ancestral EHH score below 0.05 were excluded as this threshold indicates insufficient background haplotype decay.

For *F*_ST_ values, representing genetic divergence between yak and migrated cattle populations, were calculated using the ‐‐fst command in PLINK 2.0.

Hardy–Weinberg equilibrium (HWE) was assessed within the yak population using the ‐‐hardy function in PLINK 2.0, and variants showing significant deviation (*p* < 1 × 10^−6^) were filtered out.

Nucleotide diversity (*π*) was computed for SNVs using VCFtools (v0.3.1) with a step size of 1. The difference in *π* (Δ*π* = *π*_yak_ − *π*_migrated_) served as the input for the CMS score.

Finally, the top 0.5% of SNVs (67 000 variants) exhibiting the highest CMS scores were designated as top‐scoring natural selection signals (TSNSs). Linkage disequilibrium (LD)‐based clumping was performed on these TSNSs using PLINK2 with parameters *r* ^2^ = 0.2 and a window size of 500 kb to identify independent genomic regions harboring selection signals.

Subsequently, we integrated signals from natural selection and genome‐wide association studies (GWAS) by identifying overlapping variants between the top 0.5% of CMS‐scoring SNVs and GWAS signals reaching a suggestive significance threshold (*p* < 1 × 10^−5^). This intersection yielded 6637 candidate selective single nucleotide variants, designated as Yak Selective Signals (YSSs), which implicated 172 genes. Gene‐based enrichment analysis was then performed on this set of 172 genes. The significance of enrichment was assessed using a permutation test with 10 000 iterations, and associations achieving a corrected *p*‐value < 0.0001 were considered statistically significant.

### 3.6 PDE Analysis

To quantify the contribution of yak‐selected genes (YSGs) to phenotypic divergence, we estimated the proportion of between‐population trait differences attributable to genetic variation in yak‐enriched alleles. Following previous study (Husquin et al. 2018) with modifications for allele‐frequency weighting, we calculated the Proportion of Difference Explained (PDE) as follows:

$$\mathrm{PDE}=\left[2\sum \left(\beta_{i}\times \Delta p_{i}\right)\right]/\Delta T$$

where *β*_*i*_ is the effect size of the *i*‐th yak‐enriched allele on the trait (standardized per 1‐allele copy); Δ*p*_*i*_ is the frequency difference of the *i*‐th allele between yak and migrated cattle populations; Δ*T* is the mean phenotypic trait difference between populations; ∑ is the summation across all evaluated YSG‐associated alleles.

## Author Contributions

Wangshan Zheng, Yanbin Zhu, and Xuebin Qi: conceived and designed the study. Yanbin Zhu, Xuebin Qi, and Basangwangdui: coordinated and supervised the project. Guangming Sun, Xin Li, Yanbin Zhu, Ouzhuluobu, and Xuebin Qi: collected samples and phenotype data; Guangming Sun, Xin Li, Ziqi Liu, Hui Zhang, Hui Jiang, Yiming Wang, and Siyu Ge: prepared the samples and processed to sequencing. Wangshan Zheng, and Ziqi Liu: contributed to data processing, quality control, genomic analysis, population and positive selection analysis. Wangshan Zheng, Xuebin Qi, and Yanbin Zhu: wrote and revised the manuscript. All authors read and approved the final manuscript.

## Funding

This work was supported by the China Agriculture Research System (Beef Cattle/Yak, CARS‐37), the Tibet Autonomous Region Major Special Project (XZ201901NA02, XZ202101ZD0002N‐04), Tianyou Youth Talent Lift Program of Lanzhou Jiaotong University (1520260502), the Regional Collaborative Innovation Program: Gyazakang Yak Breeding and Efficient Propagation in Linzhou County (QYXTZX‐LS2020‐01), the Open Project Program of State Key Laboratory of Barley and Yak Germplasm Resources and Genetic Improvement, Tibet Academy of Agricultural and Animal Husbandry Sciences (TAAAS) (XZNKY‐CZ‐2022‐016‐09), the Noncommunicable Chronic Diseases‐National Science and Technology Major Project (2025ZD0551803), Lhasa Municipal Major Science and Technology Project (LSKJ202508).

## Conflicts of Interest

The authors declare no conflicts of interest.

## Supporting information


**Data S1:** age70175‐sup‐0001‐Supinfo1.docx.

**Data S2:** age70175‐sup‐0002‐Supinfo2.xlsx.

## Data Availability

The data reported in this paper have been deposited in the Genome Sequence Archive (GSA: https://ngdc.cncb.ac.cn) in National Genomics Data Center Beijing Institute of Genomics, Chinese Academy of Sciences and China National Center for Bioinformation, under Bioproject: PRJCA029488. All software packages and tools employed are detailed in the Section 3.

## References

[age70175-bib-0001] Anderson, C. A. , F. H. Pettersson , G. M. Clarke , L. R. Cardon , A. P. Morris , and K. T. Zondervan . 2010. “Data Quality Control in Genetic Case–Control Association Studies.” Nature Protocols 9, no. 5: 1564–1573. 10.1038/nprot.2010.116 PMC3025522 21085122

[age70175-bib-0002] Gao, X. , S. Wang , Y. F. Wang , et al. 2022. “Long Read Genome Assemblies Complemented by Single Cell RNA‐Sequencing Reveal Genetic and Cellular Mechanisms Underlying the Adaptive Evolution of Yak.” Nature Communications 1, no. 13: 4887. 10.1038/s41467-022-32164-9 PMC9448747 36068211

[age70175-bib-0003] Gilbert‐Kawai, E. T. , J. S. Milledge , M. P. Grocott , and D. S. Martin . 2014. “King of the Mountains: Tibetan and Sherpa Physiological Adaptations for Life at High Altitude.” Physiology (Bethesda, Md.) 6, no. 29: 388–402. 10.1152/physiol.00018.2014 25362633

[age70175-bib-0004] Gou, X. , Z. Wang , N. Li , et al. 2014. “Whole‐Genome Sequencing of Six Dog Breeds From Continuous Altitudes Reveals Adaptation to High‐Altitude Hypoxia.” Genome Research 8, no. 24: 1308–1315. 10.1101/gr.171876.113 PMC4120084 24721644

[age70175-bib-0005] Grossman, S. R. , I. Shlyakhter , E. K. Karlsson , et al. 2010. “A Composite of Multiple Signals Distinguishes Causal Variants in Regions of Positive Selection.” Science 5967, no. 327: 883–886. 10.1126/science.1183863 20056855

[age70175-bib-0006] He, Y. , Y. Guo , W. Zheng , et al. 2023. “Polygenic Adaptation Leads to a Higher Reproductive Fitness of Native Tibetans at High Altitude.” Current Biology 19, no. 33: 4037–4051.e4035. 10.1016/j.cub.2023.08.021 37643619

[age70175-bib-0007] He, Y. , W. Zheng , Y. Guo , et al. 2023. “Deep Phenotyping of 11,880 Highlanders Reveals Novel Adaptive Traits in Native Tibetans.” iScience 9, no. 26: 107677. 10.1016/j.isci.2023.107677 PMC10481350 37680474

[age70175-bib-0008] Husquin, L. T. , M. Rotival , M. Fagny , et al. 2018. “Exploring the Genetic Basis of Human Population Differences in DNA Methylation and Their Causal Impact on Immune Gene Regulation.” Genome Biology 1, no. 19: 222. 10.1186/s13059-018-1601-3 PMC6299574 30563547

[age70175-bib-0009] Li, M. , S. Tian , L. Jin , et al. 2013. “Genomic Analyses Identify Distinct Patterns of Selection in Domesticated Pigs and Tibetan Wild Boars.” Nature Genetics 12, no. 45: 1431–1438. 10.1038/ng.2811 24162736

[age70175-bib-0010] Liu, X. , W. Liu , J. A. Lenstra , et al. 2023. “Evolutionary Origin of Genomic Structural Variations in Domestic Yaks.” Nature Communications 1, no. 14: 5617. 10.1038/s41467-023-41220-x PMC10509194 37726270

[age70175-bib-0011] Liu, X. , S. Zhang , Z. Cai , et al. 2022. “Genomic Insights Into Zokors' Phylogeny and Speciation in China.” Proceedings of the National Academy of Sciences of the United States of America 19, no. 119: e2121819119. 10.1073/pnas.2121819119 PMC9171634 35512099

[age70175-bib-0012] Liu, X. , Y. Zhang , Y. Li , et al. 2019. “EPAS1 Gain‐of‐Function Mutation Contributes to High‐Altitude Adaptation in Tibetan Horses.” Molecular Biology and Evolution 11, no. 36: 2591–2603. 10.1093/molbev/msz158 PMC6805228 31273382

[age70175-bib-0013] Ma, X. , C. Jia , D. Fu , et al. 2019. “Analysis of Hematological Traits in Polled Yak by Genome‐Wide Association Studies Using Individual SNPs and Haplotypes.” Genes 6, no. 10: 463. 10.3390/genes10060463 PMC6627507 31212963

[age70175-bib-0014] Peng, Y. , C. Cui , Y. He , et al. 2017. “Down‐Regulation of EPAS1 Transcription and Genetic Adaptation of Tibetans to High‐Altitude Hypoxia.” Molecular Biology and Evolution 4, no. 34: 818–830. 10.1093/molbev/msw280 PMC5400376 28096303

[age70175-bib-0015] Qi, X. , C. Cui , Y. Peng , et al. 2013. “Genetic Evidence of Paleolithic Colonization and Neolithic Expansion of Modern Humans on the Tibetan Plateau.” Molecular Biology and Evolution 8, no. 30: 1761–1778. 10.1093/molbev/mst093 23682168

[age70175-bib-0016] Qiu, Q. , L. Wang , K. Wang , et al. 2015. “Yak Whole‐Genome Resequencing Reveals Domestication Signatures and Prehistoric Population Expansions.” Nature Communications 6: 10283. 10.1038/ncomms10283 PMC4703879 26691338

[age70175-bib-0017] Qiu, Q. , G. Zhang , T. Ma , et al. 2012. “The Yak Genome and Adaptation to Life at High Altitude.” Nature Genetics 8, no. 44: 946–949. 10.1038/ng.2343 22751099

[age70175-bib-0018] Storz, J. F. 2021. “High‐Altitude Adaptation: Mechanistic Insights From Integrated Genomics and Physiology.” Molecular Biology and Evolution 7, no. 38: 2677–2691. 10.1093/molbev/msab064 PMC8233491 33751123

[age70175-bib-0019] Szpiech, Z. A. 2024. “Selscan 2.0: Scanning for Sweeps in Unphased Data.” Bioinformatics 1, no. 40: btae006. 10.1093/bioinformatics/btae006 PMC10789311 38180866

[age70175-bib-0020] Wu, T. , and B. Kayser . 2006. “High Altitude Adaptation in Tibetans.” High Altitude Medicine & Biology 3, no. 7: 193–208. 10.1089/ham.2006.7.193 16978132

[age70175-bib-0021] Xiang, K. , Ouzhuluobu , Y. Peng , et al. 2013. “Identification of a Tibetan‐Specific Mutation in the Hypoxic Gene EGLN1 and Its Contribution to High‐Altitude Adaptation.” Molecular Biology and Evolution 8, no. 30: 1889–1898. 10.1093/molbev/mst090 23666208

[age70175-bib-0022] Yang, D. , Y. Peng , Ouzhuluobu , et al. 2016. “HMOX2 Functions as a Modifier Gene for High‐Altitude Adaptation in Tibetans.” Human Mutation 2, no. 37: 216–223. 10.1002/humu.22935 26781569

[age70175-bib-0023] Zhang, H. , Y. He , C. Cui , et al. 2017. “Cross‐Altitude Analysis Suggests a Turning Point at the Elevation of 4,500 m for Polycythemia Prevalence in Tibetans.” American Journal of Hematology 9, no. 92: E552–E554. 10.1002/ajh.24809 28589696

[age70175-bib-0024] Zhang, S. , W. Liu , X. Liu , et al. 2021. “Structural Variants Selected During Yak Domestication Inferred From Long‐Read Whole‐Genome Sequencing.” Molecular Biology and Evolution 9, no. 38: 3676–3680. 10.1093/molbev/msab134 PMC8382902 33944937

[age70175-bib-0025] Zheng, W. , Y. He , Y. Guo , et al. 2023. “Large‐Scale Genome Sequencing Redefines the Genetic Footprints of High‐Altitude Adaptation in Tibetans.” Genome Biology 1, no. 24: 73. 10.1186/s13059-023-02912-1 PMC10099689 37055782

[age70175-bib-0026] Zhou, X. , and M. Stephens . 2012. “Genome‐Wide Efficient Mixed‐Model Analysis for Association Studies.” Nature Genetics 7, no. 44: 821–824. 10.1038/ng.2310 PMC3386377 22706312

