# Supplementary material for: Polygenic and Pleiotropic Adaptation of Yaks to High Altitudes: A Genome‐Wide Study of Hematological and Physiological Traits
Source: Anim Genet. 2026 Aug 11;57(4):e70175. doi: 10.1002/age.70175 (PMC13459750; doi:10.1002/age.70175)
Supplement: Supplementary file 1 [file AGE-57-0-s001.docx]

**Polygenic and Pleiotropic Adaptation of Yaks to High Altitudes: A Genome-Wide Study of Hematological and Physiological Traits**

Guangming Sun^1,2*^, Ziqi Liu^1,4*^, Hui Zhang^4,5*^, Xin Li^1*^, Ouzhuluobu^5^, Xuebin Qi^1,4,5^, Basangwangdui^1,2^, Siyu Ge^3^, YimingWang^3^, Wangshan Zheng^3*^, Yanbin Zhu^1,2*^

^1^Institute of Animal Husbandry and Veterinary Medicine, Xizang Academy of Agricultural and Animal Husbandry Sciences, Lhasa 850009, China;

^2^Key Laboratory of Animal Genetics and Breeding on Tibetan Plateau, Ministry of Agriculture and Rural Affairs, Lhasa 850000, China;

^3^School of Biological and Pharmaceutical Engineering, Lanzhou Jiaotong University, Lanzhou, Gansu, China;

^4^Kunming University of Science and Technology, Principal Investigator for Genetic Analysis and Sequencing Platform State Key Laboratory of Primate Biomedical Research (LPBR), Kunming, 650500 China;

^5^High Altitude Health Science and Translational Medicine Institute, Xizang Fukang Hospital, Lhasa 850000.

^*^These authors contributed equally to this work.

^#^Corresponding authors: Wangshan Zheng [zhengwangshan0416@163.com](mailto:zhengwangshan0416@163.com); Yanbin Zhu [zhuyanbin126@126.com](mailto:zhuyanbin126@126.com).

**Figure S1** presents a correlation analysis of yak blood phenotypes, which can be categorized into five major classes: oxygen transport (e.g., hemoglobin), coagulation function, electrolyte balance, liver function, and kidney function. These categories are consistent with the results of physiological and biochemical blood assessments**.**

**Figure S2 T**he differential analysis of 23 traits in yaks. Notably, no significant differences were detected for basophilic granulocytes (Bas) and the percentage of basophilic granulocytes (Bas%). Consequently, this figure displays the remaining 21 traits.

**Figure S3** the principal component analysis (PCA) for domestic yaks and migrated cattle (n =15 ).

 **Figure S4** The genome-wide mutation statistics and principal component analysis (PCA) for yaks. A. We identified 32,514,937 variants. B. After stringent quality control, 104 individuals and 12,855,162 variants were included in the genome-wide association studies (GWAS).

**Figure S5** presents the CMS Manhattan plot for yaks. The top 0.5% of SNVs with the highest CMS scores (CMS=8.9) were designated as top signal nucleotide variants (TSNSs). Linkage disequilibrium (LD)-based clumping was performed using PLINK to identify independent signal regions (r² = 0.2, window size = 500 kb). Different colors represent distinct GWAS signals (p<1x10^-5^).

**Figure S6** Quantile-quantile (QQ) plots for key phenotypes. (A) Hemoglobin gamma chain (HBG), (B) Mean corpuscular volume (MCV), © Ultraviolet sensitivity (UV), (D) Neutrophil count (Neu), (E) Monocyte count (Mon), (F) Platelet count (PLT), (G) Red cell distribution width coefficient of variation (RDWCV), (H) Alanine aminotransferase (ALT), (I) Triglycerides (TG).

**Figure S7** Manhattan plots of genome-wide association studies (GWAS) for key hematological phenotypes. Hemoglobin (HGB), Lymphocyte percentage (Lym%).

**Figure S8** Manhattan plots of genome-wide association studies (GWAS) for key hematological phenotypes (TIBL,UA andUREA).

**Figure S9** Manhattan plots of genome-wide association studies (GWAS) for key hematological phenotypes (TG,Mon and TP).
